# A Novel Method for the Synthesis of Tin(II) Sulphide Using Tin(II) Sulphate Precursor via H_2_-Mediated Ultrasonic Spray Pyrolysis

**DOI:** 10.3390/ma18245497

**Published:** 2025-12-06

**Authors:** Hanwen Chung, Srecko Stopic, Bernd Friedrich

**Affiliations:** IME Process Metallurgy and Metal Recycling, RWTH Aachen University, 52056 Aachen, Germany; bfriedrich@ime-aachen.de

**Keywords:** SnS, ultrasonic spray pyrolysis, hydrogen reduction, SnSO_4_

## Abstract

**Highlights:**

**What are the main findings?**

- SnS successfully synthesised via ultrasonic spray pyrolysis and H_2_ reduction.
- Unique synthesising method that is not replicable via simple solid-gas reaction.
- Thermochemical calculations of the hydrogen reduction of SnSO_4_.
- SnSO_4_ precursor enables clean, single-step conversion without substrate deposition.
- XRD confirmed SnS formation with minor SnO_2_ under 600–800 °C conditions.

**What are the implications of the main findings?**

- Demonstrates novel powder synthesis for SnS materials.
- Offers alternatives to conventional thin-film deposition routes.
- Provides insight into phase evolution during SnSO_4_-H_2_ reduction.
- Simplicity and controllable conversion route.

**Abstract:**

This study presents a novel approach for the synthesis of tin(II) sulphide (SnS) by integrating ultrasonic spray pyrolysis (USP) with hydrogen reduction (HR), using tin(II) sulphate (SnSO_4_) as a precursor. The method combines aerosol droplet generation using ultrasonic atomisation at 1.7 MHz with gas-phase reduction in a tube reactor under H_2_-N_2_ mixed gas flow. Thermochemical assessment indicated that SnS formation is thermodynamically favourable from 400 to 1000 °C, in reasonable agreement with experimental results. XRD analysis confirmed the formation of SnS as the main phase accompanied by SnO_2_ as a secondary product without SnSO_4_ when conducting USP-HR at 1000 °C. SEM images revealed flake-like, spherical, and agglomerated morphologies, with EDS confirming distinct Sn-S regions. This study demonstrates the feasibility of producing SnS powder using a simple precursor system and a clean reducing environment. The process offers a scalable and controllable synthesis route for SnS materials, providing an alternative to conventional substrate-based deposition techniques. Further optimisation of reaction temperature and residence time is expected to enhance phase purity and reduce agglomeration.

## 1. Introduction

As global demand for clean energy continues to rise, significant research efforts have been directed toward advancing semiconducting materials for use in photovoltaic and thermoelectric technologies. Some widely studied materials are, for example, cadmium telluride (CdTe) [1] and copper indium gallium selenide (CIGS) or gallium arsenide (GaAs), used in thin film solar absorbers materials for generating environmentally friendly electricity. Bismuth telluride and selenide (Bi_2_Te_3_ and Bi_2_Se_3_) [1], lead tellurides (PbTe), and Mg_2_(Si,Sn,Ge) [2] are further examples of thermoelectric materials potentially used to regenerate electricity from waste heat [3]. Some considerations that arise in these fields of materials are the hazards concern of some elements and the natural abundance of them. Elements like Cd, As, Te, and Se are all potentially biologically and ecologically toxic. Additionally, the production of such elements is closely related to the zinc and copper production industry, as they are often refined from by-products of ore processing in the primary metallurgy [1,4].

These considerations have shifted attention to possible alternatives, and one of them is tin(II) sulphide (SnS). SnS has been evaluated as a safe and cheap material in toxicity terms for Sn and S, as well as its abundance. SnS’s properties in thermoelectric and photovoltaic applications have also been widely reported [5,6,7]. These revealed the interesting opportunity and potential demand of SnS in substituting some of the above-mentioned materials. The uses of SnS extends beyond photovoltaics and thermoelectric materials into the applications of rechargeable Li-ion and Na-ion batteries [8,9,10]. Due to these, the SnS market has been predicted to experience an annual growth rate of 12.7% from 2025 to 2032, expecting to reach USD 2.5 billion by then [11]. This significant growth factor for SnS lies in its strong presence in the automotive industry, specifically for electric vehicles and hybrid electric vehicles that requires advances in battery technologies.

Regionally, the Asia-Pacific has multiple key players in SnS production such as Changsha Huajing Powdery Material (Changsha, China), ShenZhen 6Carbon Technology (Shenzhen, China), Ganzhou Orange New Materials (Ganzhou, China), ChemWill Asia (Shanghai, China), Nihon Seiko Co., Ltd. (Tokyo, Japan), and Dowa and Metals & Mining Co., Ltd. (Tokyo, Japan). Given the current dominance of the Asia-Pacific region in SnS production, exploring scalable synthesis routes and reactor designs suitable for production in other regions is becoming increasingly interesting.

### 1.1. Production of SnS

The most straightforward method to synthesise SnS powder is by reacting metallic Sn with S at elevated temperatures between 300 °C and 600 °C under conditions that suppress oxidation, such as using inert gas (e.g., Ar) or using graphite or iodine [5,12], forming multi-crystalline SnS according to the following reaction:

Sn + S +(C,Ar,I_2_) → SnS + (C,Ar,I_2_) (1)

The importance of preventing oxidation phenomena during this synthesis route is to prevent the formation of unwanted tin(IV) sulphide, SnS_2_. Other possible forms of tin-sulphur compound is tin(II,IV) sulphide, Sn_2_S_3_. SnS generally exhibits p-type semiconductor behaviour, while both SnS_2_ and Sn_2_S_3_ have been reported to be n-type semiconductors [13].

Burton et al. [14] conducted chemical vapour transport (CVT) to produce macroscopic crystals of SnS using Sn powder (>99 wt.%) with I_2_ pieces and S pieces (both >99.999 wt.%) at 850–950 °C. Due to the aim of achieving single-crystal growth, the reaction was maintained for 10 days. The results have confirmed the presence of SnS, despite the coexistence of SnS_2_ that reduces the efficiency of SnS as a photovoltaic absorber layer. The authors concluded the importance of careful stoichiometric control for the ratio of Sn:S to prevent the formation of alternative S-rich phases. Other methods that uses Sn and S as precursors have also been reported in [15,16]. In addition to using elemental precursors, there are possibilities of using organic complexes as precursors via aerosol-assisted chemical vapour deposition (AACVD), a few of which were introduced in detail by the review from Norton et al. [5]. The possibilities of using SnCl_2_ and H_2_S as precursors were reported by Price et al. [17] reported that using SnCl_2_ and H_2_S as precursors at 545 °C produced single-phase dark grey SnS film without other phases.

Among the methods discussed, the resulting SnS is generally produced in bulk form, meaning as a three-dimensional material. The behaviour of two-dimensional (2D) nanoscale SnS generally exhibit drastically different properties compared to its bulk form, such as optoelectronic properties [18]. Furthermore, two-dimensional SnS has a high level of long-term cycling stability, also in acidic and electrolytic conditions, making it even more attractive for the applications mentioned so far [5].

According to Norton et al. [5], various methods have been reported for synthesising nanoscale SnS, including hydrothermal, solvothermal, aqueous solution, and hot injection methods and classified into top-down (breaking down of bulk material into nanoscale) and bottom-up methods (direct synthesise of nanomaterials). One specific method that is more relevant to this current investigation is the spray pyrolysis method.

Alam and Dutta [19] deposited SnS nanofilms on glass substrate using the continuous spray pyrolysis (CoSP) technique with SnCl_2_ and SC(NH_2_)_2_ precursors. These precursors were first dissolved in deionised water and sprayed in the CoSP reactor at a flow rate of 2–3 mL/min using N_2_ carrier gas at 6 l/min. The sprinkles of carrier gas then go through the first zone at 350 °C, the second zone at 700 °C, and finally deposit on the glass substrate in the third zone at 350 °C. Although detailed reactions in the three respective zones were not reported, the reaction product deposited on the glass was shown to consist of only single-phase SnS according to XRD analysis. A similar approach was conducted by Reddy and Reddy [20], but the precursors were dissolved in isopropyl alcohol and deionised water, and temperature was kept at 300–350 °C with compressed air as a carrier gas. Calixto-Rodriguez et al. [21] modified the deposition conditions to separately deposit SnS_2_ as well as SnS. The authors mentioned the change in phases from the mixture of SnS-SnS_2_ at T = 455 °C and SnS-SnO_2_ at T = 488 °C. This phenomenon is likely due to the usage of compressed air as a carrier gas, which contains certain levels of oxidising atmosphere.

### 1.2. Ultrasonic Spray Pyrolysis (USP)

**Figure 1:**
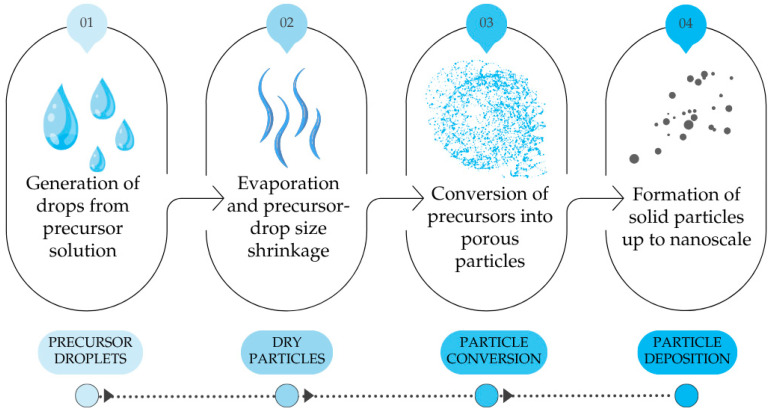
General procedure of spray pyrolysis, self-drawn according to [22].

The spray pyrolysis is a low-cost technique, simple, and has the advantage of deposition over larger areas. The general procedure of spray pyrolysis is shown in the following Figure 1.

In step 1, precursor solution droplets can be generated either through liquid atomisation by high velocity air or through ultrasonic atomisation without air. Although ultrasonic atomisation suffers from low throughput, it possesses the advantage of narrow drop size distribution and, in the case of SnS synthesis, also the absence of oxidising atmosphere from air. By altering the frequency of the atomiser, ultrasonic spray pyrolysis (USP) has the advantage of using precursor aerosol or precursor “mists” as input to the heated chamber. The application of USP for the preparation of nanoparticles has been widely reported for various types of materials including Ag, Ni, Co, TiO_2_, and ZnO [23,24,25].

From Figure 1, the carrier gas used to transport the atomised precursor solution can be either inert gas such as Ar, N_2_ that act only as a transport media, or active gas such as H_2_ to mediate certain reactions with the precursor. Stopic et al. [26] investigated the hydrogen reduction (HR) behaviour of Fe_2_O_3_ via USP for the preparation of nanoscale Si-doped and Pt-doped nanoparticles, citing the feasibility of this integrated process. Similar feasibility of USP-HR has also been reported by Choi et al. [27] for the synthesis of W/Y_2_O_3_ powders from WO_3_/Y_2_O_3_ and by Gurmen et al. [28] for the synthesis of Fe-Ni alloy from FeCl_2_ and NiCl_2_.

Due to the effective combination of USP-HR, this study aims to present a novel method for the production of SnS nano-powders. For this purpose, tin(II) sulphate (SnSO_4_) is selected as a precursor. Using SnSO_4_ for the synthesis of SnS is not entirely new, as reported by Kumar [29] and Ghosh et al. [30], using Na_2_S for S supply in the SILAR process, and Kamel and Ibrahim [31], using Na_2_SO_4_ for S supply in the electrochemical co-deposition of Sn and S. This precursor can also be used to synthesise SnS_2_ as reported by Kamkui et al. [32], who also suggested a series of governing equations according to Lux-Flood’s base:

(2) $${\mathrm{SnSO}}_{4}\;\overset{Dissolve\;in\;S_{Liquid}}{\to }{\mathrm{Sn}}^{2+}+{{\mathrm{SO}}_{4}}^{2-}$$

(3) $${{\mathrm{SO}}_{4}}^{2-}\overset{Dissolve\;in\;S_{Liquid}}{\to }{\mathrm{SO}}_{3}+0.5O_{2}+2e^{-}$$

S + 2e^−^ → S^2−^ (4)

Sn^2+^ + S^2−^ → SnS (5)

SnS + S → SnS_2_ (6)

The mechanism proposed involves the formation of SnS but as an intermediate species instead of a final product. In these steps, the oxidation of O^2−^ to O_2_ and reduction in S to S^2−^ is the key step, which enables the formation of SnS and eventually SnS_2_. By accurately controlling the S supply within the system and ensure a reducing atmosphere, it is possible to produce SnS.

To prevent the oversupply of S in the system, this study uses only SnSO_4_ as a precursor and H_2_ as a reducing agent. This study primarily aims to establish the proof of concept for the synthesis of SnS using the proposed method. Accordingly, achieving a product composed of multiple Sn_x_S_y_ phases is acceptable as long as it confirms the successful synthesis of SnS through this approach. Being a novel method, this study will also present a thermochemical evaluation of the reaction and thermal decomposition of SnSO_4_ under inert atmosphere. The effects of the parameter combinations on the form products will be characterised by X-ray diffraction (XRD) and a scanning electron microscope with energy-dispersive spectroscopy (SEM-EDS).

## 2. Materials and Methods

Prior to the experimental trials, the thermochemical modelling software FactSage 8.3 (GTT Technologies, Herzogenrath, Germany) was used to conduct preliminary feasibility calculations of the reaction between SnSO_4_ and H_2_, as well as the possible product formation. Furthermore, the thermal decomposition of SnSO_4_ under inert atmosphere was investigated using DSC analysis (Netzsch STA 449F3, Netzsch Group, Selb, Germany) to confirm endotherm/exothermic events, as well as to understand possible side reactions happening to the decomposed product inside the heated tube furnace. This study was conducted under Ar atmosphere from 25 °C to 800 °C at heating rates of 2 K/min and 10 K/min. This is deemed necessary, as the precursor solution will pass through a heated tube furnace, where both the hydrogen reduction and the thermal decomposition could take place.

For the experiment trials, synthetic as-purchased SnSO_4_ powders (96% min from Thermo Fisher Scientific, Waltham, MA, USA) were used as a precursor. The experimental procedure starts with characterising the initial material’s chemical composition using ICP-OES (Spectro Arcos, SPECTRO Analytical Instruments Gmb, Kleve, Germany), shown in the following Table 1.

Furthermore, the retention time of the droplets in the reaction tube can be calculated by relating the volume of heating zone in the tube, *V*, the flow rate of the droplets, *q*, the reaction temperature, *T_r_,* and room temperature, *T*_0_, according to the following equation.

(7) $$t=(\frac{V\cdot T_{0}}{q\cdot T_{r}})$$

### Experimental Procedure and Setup

**Figure 2:**
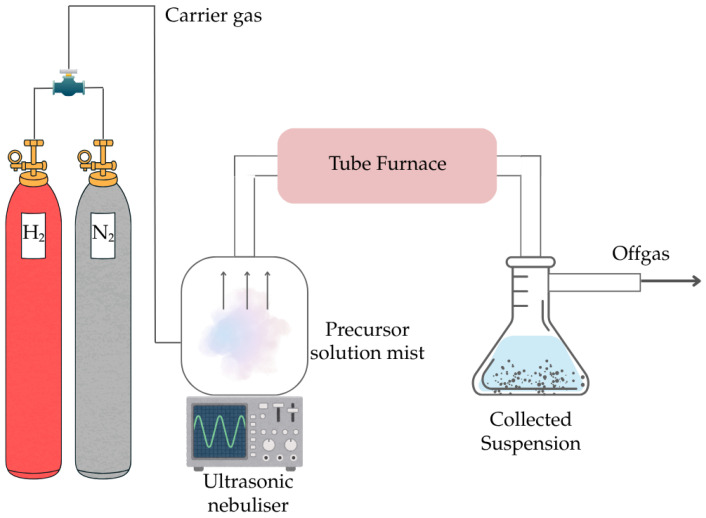
Schematic setup of USP-HR used in this study.

The as-purchased SnSO_4_ is first dissolved in deionized water to prepare 0.5 M of precursor solution and added to the ultrasonic nebuliser (PRIZNano, Kragujevac, Serbia) shown in the experimental setup is (Figure 2). The tube furnace (Ströhlein, Selm, Germany) with a quartz glass tube was first heated to the investigated temperature (T = 400, 600, 800, 1000 °C). This quartz glass tube has a heating zone of 55 cm length and 1.05 cm diameter. The ultrasonic nebulizer that has three transducers with a frequency of 1.75 Mz generates the precursor droplets (aerosol) once the target temperature has been reached in the tube furnace. The aerosol was carried with a mixed gas of 0.5 L/min N_2_ and 1.5 L/min H_2_ into the quartz tube. After the investigated holding time of 1 hr, the solid deposits were filtered from the collected suspension in the precipitator bottle and further processed by drying at 105 °C for 2 h to obtain the final product. The product was then analysed with SEM (JSM 7000F by JEOL 2006, JEOL Ltd., Tokyo, Japan), EDX (Octane Plus-A by Ametek-EDAX construction year 2015, AMETEK Inc., Berwyn, PA, USA), and XRD (Bruker D8 Advance, Karlsruhe, Germany) with Mo wavelength of 0.71Å. All XRD results were analysed using HighScorePlus (Malvern Panalytical GmbH, Kassel, Germany), with background correction using the Sonneveld-Visser method (bending factor = 6, granularity = 21), followed by smoothing (smoothing level = 5). All samples were measured under identical instrument settings, and only experimental data is presented in this work. A list of experimental trials with corresponding parameter is compiled in Table 2.

**Figure 3:**
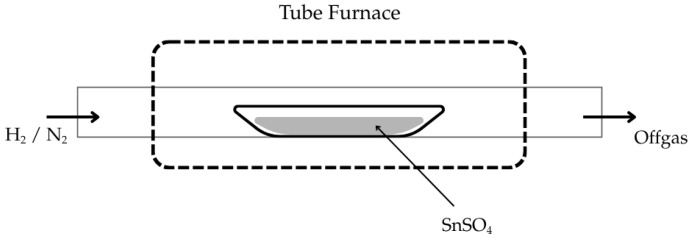
Schematic setup of fixed-bed experiment used in this study.

To demonstrate that this reaction could only work under USP, repeated experimental trials with similar parameters were conducted in an isothermal fixed-bed setup (compare Figure 3). Here, 2 g of SnSO_4_ powder was placed on a crucible boat inside the same tube furnace. The temperature was increased from room temperature at a heating rate of 600 K/h with a constant H_2_ gas flow of 1 L/min. After a holding time of 1 h at the selected temperature, the H_2_ gas supply was switched off and replaced with N_2_ gas flow of 1 L/min until the furnace reached room temperature. The reaction products were then analysed by XRD.

## 3. Results

### 3.1. FactSage Thermochemical Calculation

**Figure 4:**
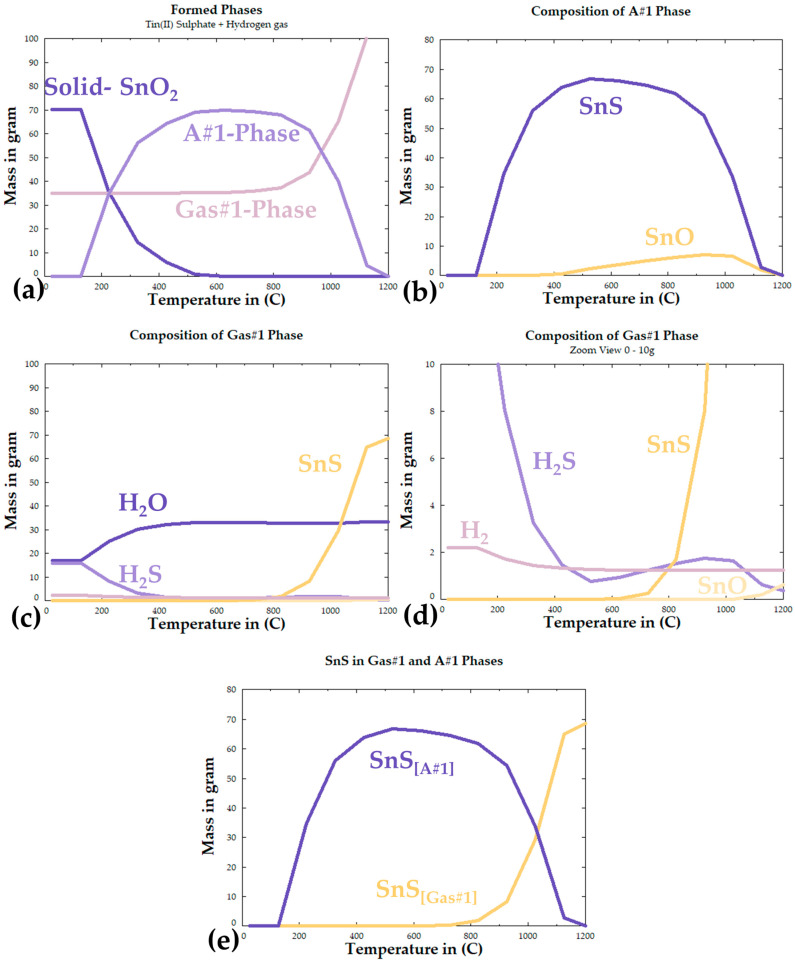
FactSage calculated reaction products: (**a**) total formed phases, (**b**) composition of A#1 phase, (**c**) composition of Gas#1 phase, (**d**) zoomed view of Gas# 1phase composition, (**e**) overall formation of SnS in both phases.

According to the chemical impurities of the as-purchased SnSO_4_ shown in Table 1, impurities are in ppm range and should not contribute to any side reactions in the process. Therefore, the thermochemical calculation is performed by using 100% SnSO_4_. By using the *Equilib* function with the database of FTOxid, FTSalt, FTSulf, and FactPS, the temperature-dependent product formation is calculated from room temperature up to 1200 °C, and the results are shown in Figure 4. The results have revealed the formation of three phases: a solid SnO_2_ phase, Gas#1 phase, and A#1 phase. Under perfect thermochemical conditions, the first phase to form when SnSO_4_ reacts with H_2_ is SnO_2_ and H_2_S, taking place up to ~120 °C. The SnO_2_ and H_2_S react to form SnS from 120 °C up to ca. 500 °C, where all the SnO_2_ and H_2_S are “consumed”. According to the diagram, the optimal temperature for the maximum formation amount of solid SnS is at 539.31 °C at 95.998% SnS and 4.0017% SnO (compare peak SnS mass in Figure 4e). Despite having a melting point at 882 °C and boiling point at 1230 °C, the amount of SnS in the A#1 phase can be seen decreasing from that peak temperature, moving into the Gas#1 phase together along the formation of SnO in both the A#1 phase and the Gas#1 phase. The presence of SnO at higher temperatures could be explained by the oxidation of the SnS by water vapour. At higher temperatures above ~1150 °C, it can be seen that all SnS should be present in the Gas#1 phase with the presence of SnO. Specifically, the calculation shows a gas phase that contains 62.8% H_2_O, 21% H_2_, 15.46% SnS, and the rest of SnO and H_2_S. According to these calculations, it can be deduced that the reaction temperature suitable for the hydrogen reduction in SnSO_4_ lies within the range of 400–1000 °C.

Although the FactSage results provide an important forecast about possible reaction products, the thermochemical calculation considers ideal and favourable conditions within an enclosed system where all phases are in equilibrium. Considering the real reaction conditions, i.e., constant flow of carrier gas in the USP-HR process, the specific reactions and reaction products would come in different orders and intensities. Furthermore, the calculations do not consider the kinetics condition. As the SnSO_4_ in this study is present in the reaction chamber in the form of dissolved, fine droplets generated by an ultrasound nebuliser, a completely different reaction through thermodynamics that is strongly kinetically improved should be expected. This briefly explains the rational of the selected temperature range 400–1000 °C, as even if the SnS goes into the Gas#1 phase, the gas will still pass through the precipitator bottle, where rapid cooling would likely lead to precipitation of the products.

**Figure 5:**
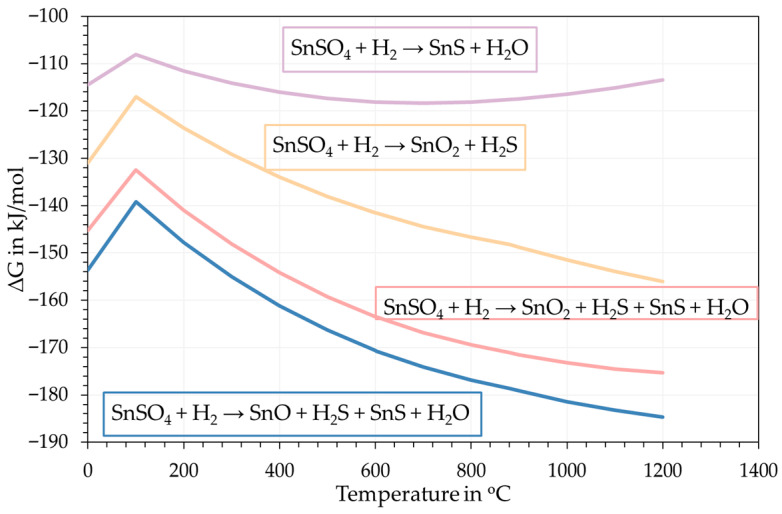
Gibbs free energy change in expected reactions taking place.

Other than the product formation, the same selected database from FactSage was also used to calculate the Gibbs free energy changes in possible reactions as shown in Figure 5. The Gibbs energy changes in the reactions of interests have negative value from room temperature to 1200 °C, which confirms a high possibility for the formation of SnS through H_2_ reduction of SnSO_4_, albeit with the co-formation of Sn-O phases according to these calculations.

### 3.2. DSC Analysis

**Figure 6:**
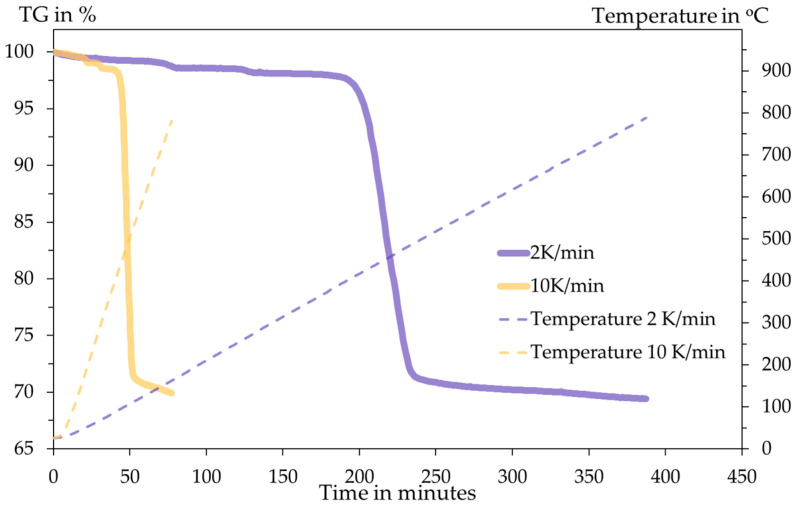
Thermogravimetry diagram and temperature programme with respect to time.

**Figure 7:**
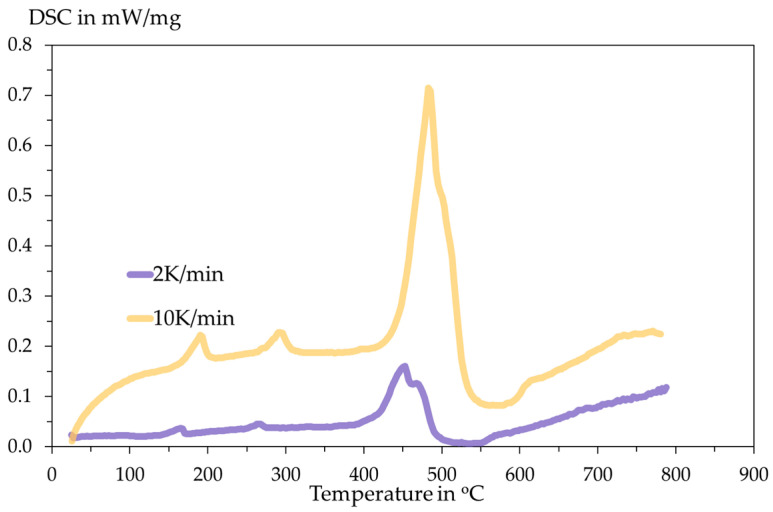
Recorded DSC signals with respect to temperature.

The DSC analysis is conducted inside an Al_2_O_3_ crucible with an initial sample mass of 14.96 mg for 2 K/min and 11.35 mg for 10 K/min. The heating programme and the DSC results are shown in the following Figure 6 and Figure 7.

The DSC curves measured under inert Ar atmosphere at 2 K·min^−1^ and 10 K·min^−1^ show multi-step thermal behaviour. Small peaks appear below ≈300 °C, while the dominant decomposition event occurs in the T ≈ 450–520 °C range. These readings are significantly larger and shifted to a higher temperature at the higher heating rate. The main peak of the DSC is assigned to the thermal decomposition of SnSO_4_ with the evolution of sulphur oxides and the formation of SnO/SnO_2_. The observed peak shift and change in peak shape with heating rate indicate kinetic control of the process. The larger amplitude at 10 K/min and higher peak temperature are classical kinetic effects: at faster heating, the reaction proceeds later (shifts to higher T) and appears more intense/overlapped because multiple sub-steps cannot separate kinetically. To confirm reaction stoichiometry, XRD analysis of residues is conducted.

In both tests, the total mass losses are 30.57% and 30.08% for 2 K/min and 10 K/min, respectively. This amount of mass loss briefly indicates the release of SO_3_ gas upon heating according to the following Equation (8). The stoichiometric mass loss in this equation is 29.84%, showing close proximity to the TGA results.

SnSO_4_ → SnO_2_ + SO_3_ (8)

**Figure 8:**
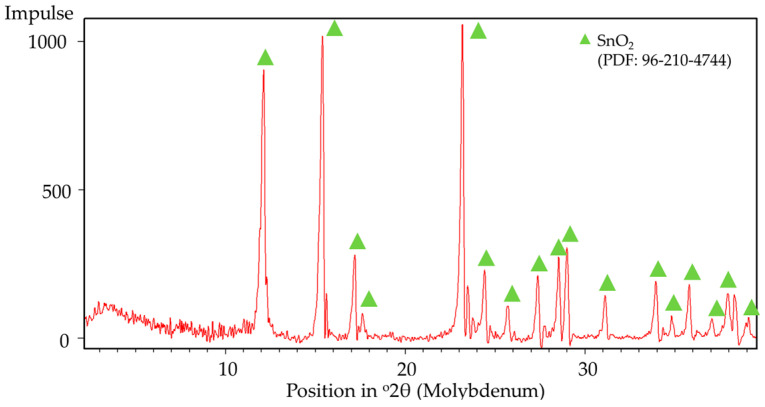
XRD analysis results of post DSC-TGA products.

**Figure 9:**
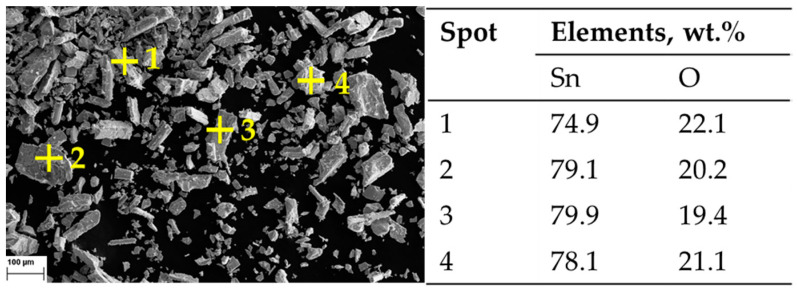
SEM Image of post DSC-TGA products: 100× magnification, with corresponding EDX measurements.

**Figure 10:**
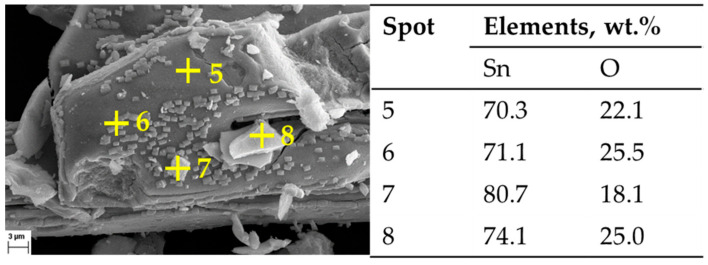
SEM Image of post DSC-TGA products: 2000× magnification, with corresponding EDX measurements.

The assumption of decomposition according to Equation (8) is confirmed by XRD analysis (Figure 8) of the products after DSC-TGA, showing only SnO_2_ phases. This is further confirmed by the SEM-EDS analysis (Figure 9 and Figure 10) that shows the ratio of Sn:O very close to SnO_2_ (theoretical amount: 78.8 wt.% Sn and 21.2 wt.% O). The low-magnification analysis revealed a homogenous distribution of Sn and O across the sampled area, as the larger interaction volume averages the signal from multiple particles. In contrast, point measurements acquired at higher magnification on individual particles showed greater deviation. This is attributed to the analysis probing particle heterogeneity, local surface topography, and orientation effects, which are magnified when the sampling volume is reduced to a single particle. Despite these local variations, which are common in semi-quantitative EDX analysis of nanomaterials, the stoichiometry is in agreement with the XRD results.

The results of the thermal decomposition show that at elevated temperature, SnSO_4_ will decompose into SO_3_ and SnO_2_. Due to this, synthesising SnS from SnSO_4_ using H_2_ as a reducing agent can only be completed if both the gas and solid are heated simultaneously. Otherwise, the formation of SnO_2_ will lead to H_2_ reacting with it, producing metallic Sn.

### 3.3. USP-HR for Synthesising SnS

**Figure 11:**
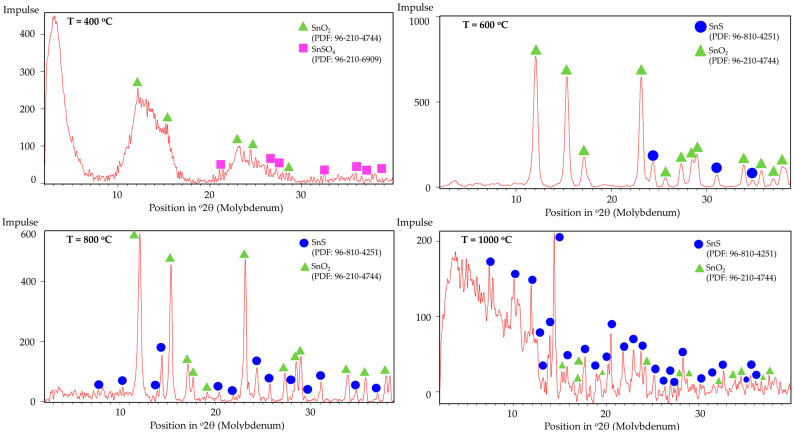
XRD peaks of USP products synthesised at T = 400, 600, 800, and 1000 °C.

The phase composition of the synthesised powders was analysed by XRD. Figure 11 shows the XRD patterns of the samples produced at different temperatures. The diffraction peaks correspond to SnSO_4_ structure, SnO_2_ structure, and SnS structure with the respective PDF numbers listed in the diagram.

**Figure 12:**
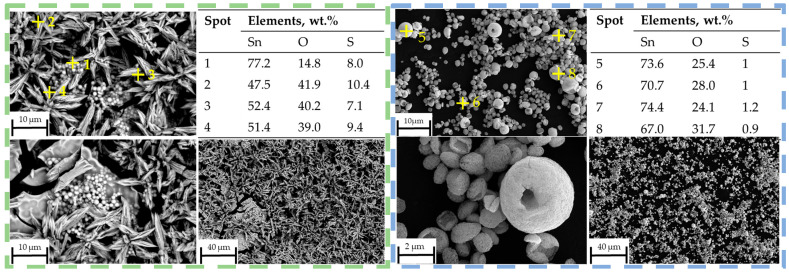
SEM images with EDS measurement spots on reaction products (green frame) at 400 °C, (blue frame) 600 °C.

**Figure 13:**
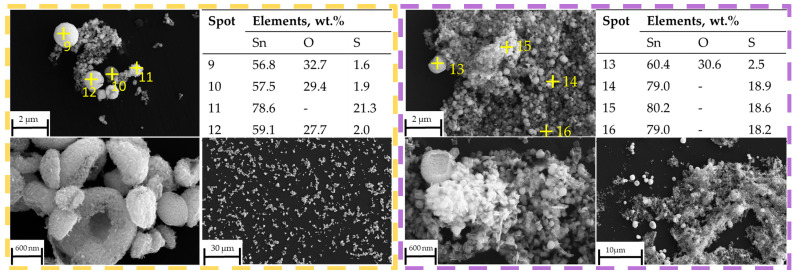
SEM images with EDS measurement spots on reaction products (yellow frame) at 600 °C, (purple frame) 1000 °C.

No obvious peaks corresponding to SnS can be identified in the XRD pattern at 400 °C, within the detection limits of the measurement, but the presence of SnO_2_ confirms that thermal decomposition of SnSO_4_ took place, even with H_2_ present in the system. However, due to the relatively short retention time within the tubular furnace, subsequent conversion of the SnO_2_ into metallic Sn was not observed. The phase identification at 400 °C is less certain due to the poorer quality of the XRD data, exhibiting features of incomplete reaction and low crystallinity. Starting from T = 600 °C, the XRD shows several peaks from SnS. Despite reflecting in the XRD peaks, EDS measurements on SEM images did not clearly indicate the presence of SnS (compare blue frame in Figure 12). At higher temperatures of 800 °C and 1000 °C, a greater presence of SnS can be seen both in XRD and SEM. Specifically, this can be visually distinguished by the spherical shape of SnO_2_ and fine platelet-like particles of SnS in the purple frame in Figure 13. It has to be mentioned that the evaluation of presence in the SEM images is made solely based on the elemental percentage of the compounds SnSO_4_, SnS, and SnO_2_ from the EDS measurements and its corresponding theoretical amount according to Table 3.

**Figure 14:**
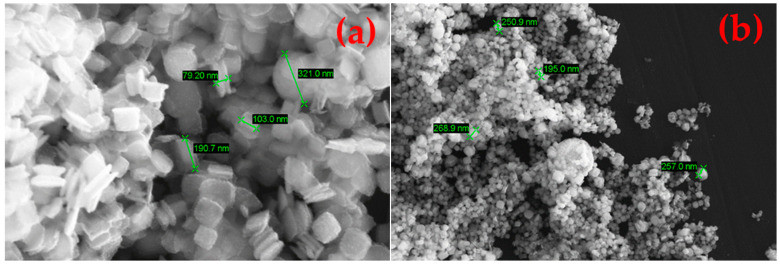
SEM images of products from USP-HR at 1000 °C showing nanoscale particles: (**a**) 50,000× zoom and (**b**) 10,000× zoom.

**Figure 15:**
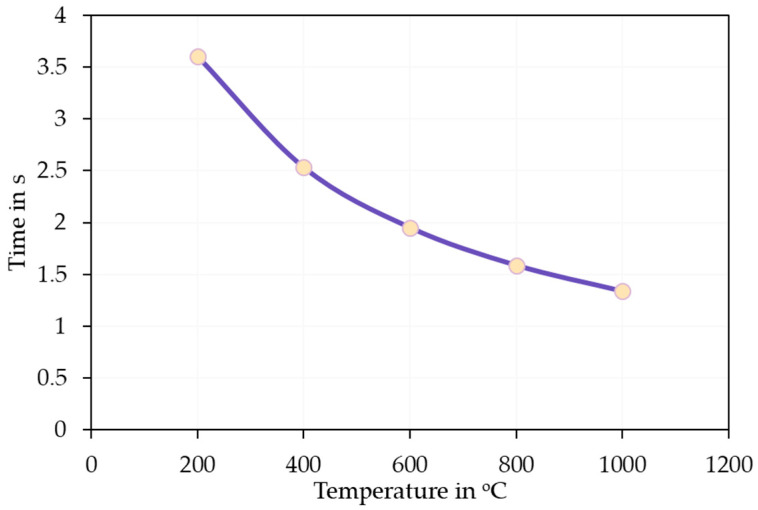
Retention time calculated according to Equation (7).

**Figure 16:**
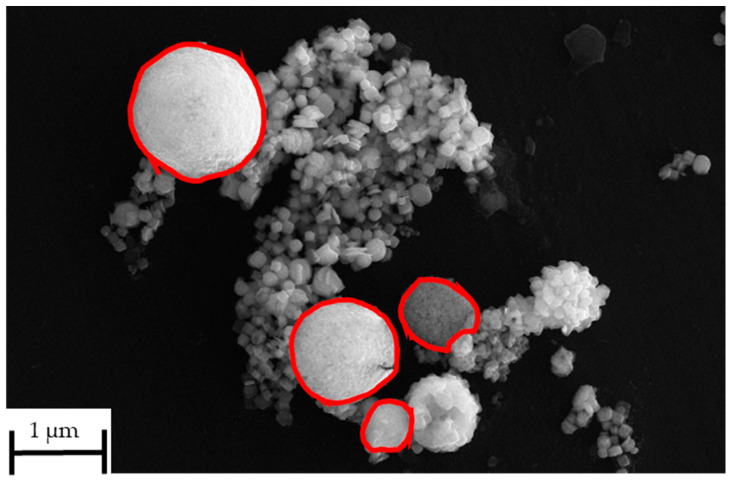
Distinction between oxide particles and SnS particles in SEM image from USP-HR at 1000 °C.

The as-synthesised SnS in the reaction product exhibits agglomerated, plate-like, and partially irregularly shaped particles with nanoscale dimensions (compare Figure 14). The individual crystallites appear to range from approximately 80 nm to 300 nm in size. These nanostructured platelets are typically seen in orthorhombic SnS [33], which forms layered morphologies likely due to its anisotropic crystal structure. The densely packed morphology makes it difficult to discern a clear particle size distribution. This can be briefly explained by the extremely short retention time of the precursor droplets according to Equation (7). On average, the droplets spent 1–3 s in the heated zone before being transported out into the precipitator bottle (compare Figure 15). The increase in the retention time, i.e., through lower flow rate or longer reaction zone, may be able to reduce agglomeration by allowing a more complete reaction. Nevertheless, a distinct difference between the SnS particles and oxide particles can be clearly observed in Figure 16 (circled in red), indicating a successful synthesis.

**Figure 17:**
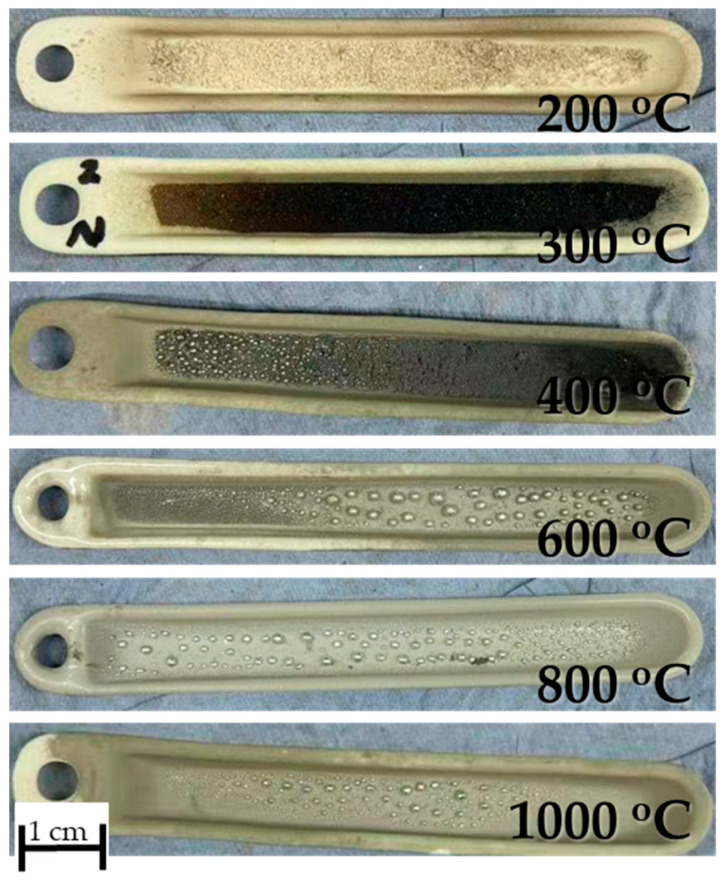
Reaction products of fixed-bed isothermal experiments from (**top**) 200 °C to (**bottom**) 1000 °C.

**Figure 18:**
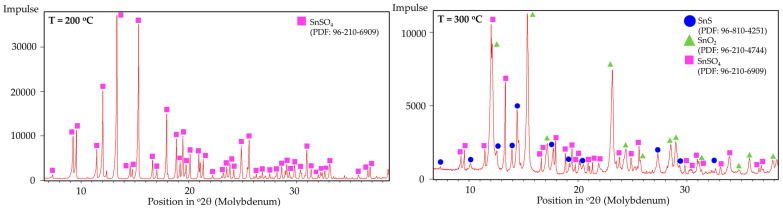
XRD peaks of fixed-bed isothermal experimental products from (**left**) T = 200 °C to (**right**) T = 300 °C.

The fixed-bed isothermal experiments have shown that the formation of SnS was not effective; instead, the formation of SnO_2_ and Sn were more prominently measured and observed. This can be explained by the poor contact between H_2_ and the solid SnSO_4_ that leads to the thermal decomposition into SnO_2_ (as briefly explained in Section 3.2 DSC analysis). Upon formation of SnO_2_, the constant H_2_ flow reduces it into metallic Sn (compare metal droplets in Figure 17). The XRD analysis of the products from T = 200 °C and 300 °C are shown in Figure 18. Corresponding well to the FactSage calculation in Section 3.1 and DSC analysis in Section 3.2, the products from T = 200 °C have shown no obvious changes in its phase composition. At 300 °C, multiple simultaneous reactions took place that led to the formation of both SnS and SnO_2_ phases, albeit the weak signals for the intended SnS phase. Samples from 400 °C onwards were not analysed since these already show metal droplets and greyish metal powder. Despite being thermochemically feasible, running the reduction process for the synthesis of SnS under standard fixed-bed isothermal conditions has been proven not effective, making the USP-HR process a unique and important advancement in the synthesis of SnS using SnSO_4_ as a precursor.

## 4. Conclusions

With the expanding interest in advance applied materials in different technological sectors, the availability of raw materials for fabrication becomes an important consideration within the supply chain. SnS has attracted attention due to its semiconducting and optoelectronic properties, making it a material of interest for further investigation. The synthesis of SnS was conventionally performed by spray pyrolysis, CVT, AACVD, and the SILAR process, which requires the deposit substrate to be present in the process. This study demonstrates the feasibility of the preparation of SnS through a novel concept of combined ultrasonic spray pyrolysis and hydrogen reduction using SnSO_4_ as a precursor.

Thermochemical evaluation confirmed that the reaction pathway is thermodynamically favourable within the temperature range of 400–1000 °C. Experimentally, the formation of SnS was confirmed at 800 and 1000 °C, with only a trace indication at 600 °C and no detection at 400 °C. Alongside SnS, SnO_2_ was also identified, likely resulting from partial oxidation of intermediate Sn species or incomplete reduction under the selected conditions.

Morphological analysis from SEM images revealed a mixture of flake-like, spherical, and agglomerated particles, suggesting rapid particle growth during droplet reaction. EDS confirmed the presence of both Sn and S, supporting the formation of SnS, while distinct regions corresponding to oxide phases were also identified. Despite the formation of mixed Sn-S-O phases, the results establish a successful proof of concept for producing SnS via an integrated USP-HR process. The approach offers advantages of clean reduction chemistry, controllable droplet generation, and potential scalability for continuous powder synthesis. Future work could focus on optimising residence time, temperature control, and post-processing to minimise oxide formation and improve product uniformity. The properties of SnS products synthesised through USP-HR should also be studied and characterised to rationalise the scaling of USP-HR.

## Figures and Tables

**Table 1:** Chemical composition of impurities in used SnSO_4_ from ICP-OES analysis (values in ppm).

Al	Cr	Cu	Fe	Nb	Ni	P	Si	Zn
<20	<10	10	50	<15	<15	<100	<50	12

**Table 2:** List of experimental trials with temperature and holding time as parameters.

Test	Temperature [°C]	Holding Time [h]	Input Amount	Method
1	400	1	0.5 M	USP-HR
2	600	1	0.5 M	USP-HR
3	800	1	0.5 M	USP-HR
4	1000	1	0.5 M	USP-HR
5	200	1	2 g	Fixed-Bed
6	300	1	2 g	Fixed-Bed
7	400	1	2 g	Fixed-Bed
8	600	1	2 g	Fixed-Bed
9	800	1	2 g	Fixed-Bed
10	1000	1	2 g	Fixed-Bed

**Table 3:** Theoretical mass percent composition of Sn, O, and S in different compounds.

Elements/Compounds	SnSO_4_	SnS	SnO_2_
Sn	55.27 wt.%	78.74 wt.%	78.75 wt.%
S	14.93 wt.%	21.26 wt.%	-
O	29.80 wt.%	-	21.23 wt.%

## Data Availability

The original contributions presented in this study are included in the article. Further inquiries can be directed to the corresponding authors.
